# Supplementary material for: Content-rich biological network constructed by mining PubMed abstracts
Source: BMC Bioinformatics. 2004 Oct 8;5:147. doi: 10.1186/1471-2105-5-147 (PMC528731; doi:10.1186/1471-2105-5-147)
Supplement: Additional File 2 — The original results of the above study (non-essential files are deleted to keep the file size under the limit set by BMC bioinformatics). [file 1471-2105-5-147-S2.bz2 › chilibotAdditionalFile2/dip10/127ID8609467E471/html/P_N.html]

 


 **P** and **N** 
  
Found 149323 abstracts in PubMed, retrieved 10.  
 

 What does Google say? 
 PDF only 
| .edu only 

---

**Interactive relationship** (e.g. stimulation, inhibition, etc)

**Non-interactive relationship** (e.g. studied together, co-existance, homology, etc.)

- The median number of days with positive blood cultures was 2 for the MRSA  **n**  = 16 and 1 for the MSSA  **n**  = 18 patients  **P**  = 0.04.  Ref: 12867833 Pediatr Infect Dis JPediatr Infect Dis J,
